# Fast Depth Estimation in a Single Image Using Lightweight Efficient Neural Network

**DOI:** 10.3390/s19204434

**Published:** 2019-10-13

**Authors:** Sangwon Kim, Jaeyeal Nam, Byoungchul Ko

**Affiliations:** Department of Computer Engineering, Keimyung University, Daegu 42601, Korea; swkim@stu.kmu.ac.kr (S.K.); jynam@kmu.ac.kr (J.N.)

**Keywords:** depth estimation, convolutional neural network, lightweight efficient neural network, model, single image, ordinal regression

## Abstract

Depth estimation is a crucial and fundamental problem in the computer vision field. Conventional methods re-construct scenes using feature points extracted from multiple images; however, these approaches require multiple images and thus are not easily implemented in various real-time applications. Moreover, the special equipment required by hardware-based approaches using 3D sensors is expensive. Therefore, software-based methods for estimating depth from a single image using machine learning or deep learning are emerging as new alternatives. In this paper, we propose an algorithm that generates a depth map in real time using a single image and an optimized lightweight efficient neural network (L-ENet) algorithm instead of physical equipment, such as an infrared sensor or multi-view camera. Because depth values have a continuous nature and can produce locally ambiguous results, pixel-wise prediction with ordinal depth range classification was applied in this study. In addition, in our method various convolution techniques are applied to extract a dense feature map, and the number of parameters is greatly reduced by reducing the network layer. By using the proposed L-ENet algorithm, an accurate depth map can be generated from a single image quickly and, in a comparison with the ground truth, we can produce depth values closer to those of the ground truth with small errors. Experiments confirmed that the proposed L-ENet can achieve a significantly improved estimation performance over the state-of-the-art algorithms in depth estimation based on a single image.

## 1. Introduction

Depth estimation from objects or scenes has been studied for a long time in the computer vision field and has been applied in various applications, such as 3D modeling, computer graphics, virtual reality, augmented reality, and autonomous driving. There are two general methods used to obtain a depth estimation from an object or real-world scene, namely, passive and active, both of which are extremely popular. With a passive method, multiple (usually two) cameras are employed to acquire 3D coordinates of the target 3D scene. Parallax, intrinsic, and extrinsic characteristics of the cameras provide the depth of the scenes and 3D real-world coordinates [1]. This method uses 3D depth information predicted by applying stereo matching to images obtained through the simultaneous capturing of objects using two or more cameras. The method is relatively inexpensive and can be used in outdoor environments, unlike methods applying an infrared sensor; however, the computational time required for matching is longer. In addition, it is difficult to generate accurate depth information when the distance between the cameras and the angle are misaligned [2,3].

With an active method, structured light (SL) [4], shape from shading (SFS) [5], and shape from texture (SFT) [6] methods are employed to acquire 3D information of the target 3D scene. This approach calculates the depth and surface information of the objects through analyzing the observed light patterns, brightness of one black and white, and textured surface in the projected images. However, the reconstruction results of this approach are not precise under complex lighting or texture conditions such as in outdoor scenes.

Another widely employed active method is time-of-flight (ToF) [7], which estimates the depth of a target scene based on the known information from the speed of light projected onto a target scene [8]. In addition, RGB-D cameras, which a specific type of depth sensing devices have received significant attention with appearance of Microsoft Kinect 1. The upgraded version of Kinect1, Kinect 2, has the advantage of supporting higher resolution than Kinect 1. Similarly, Intel RealSense calculates depth using a pair of depth sensors, an RGB sensor, and an infrared projector. To generate a depth map, these sensors emit infrared rays and the patterns of the rays reflected from the object are detected to determine the curvature and distance. Infrared sensors are, however, more expensive than cameras and the space for such installations is limited. Moreover, because of the nature of infrared rays, the performance is in general degraded in outdoor environments where the natural light is strong. In addition, because the radiation distance of infrared rays is limited, infrared sensors can be used only for the estimation of a near object.

To mitigate any problems from a practical perspective, a single-image-based depth map estimation is considered one of the most reliable alternatives to existing approaches, including the use of stereo vision, SL, SFT, SFT, ToF, and RGB-D sensors or their convergence. Several approaches have been proposed to extract depth maps using a single image. A popular method is to quantify the lens properties for the focus and defocus and to estimate the 3D depth based on the concept of learning-based technologies, including neural networks and deep learning.

Recently, as the field of artificial neural networks (ANNs) has rapidly developed, research studies on estimating 3D information from images have been actively conducted. In particular in the computer vision field, ANN learning together with the convolutional neural networks (CNNs) that have emerged allow the recognition of the semantic information and perspective of the objects of a single image [9,10,11,12,13]. In other words, the fact that it is possible to extract various features and recognize scale invariance features from an image by applying convolution operation in multiple layers of the neural network allows a depth map to be estimated using only a single image by learning various scenes and depth information in advance. Such a single image-based depth map estimation offers an advantage in that the time required for camera calibration and image matching can be reduced by constructing a 3D depth map using a single image without using a stereo image or multi-view images.

Accurate depth map estimation can be achieved through pixel-by-pixel prediction. To achieve this, dense feature maps should be extracted using a relatively large number of parameters in each layer of the CNN, or sparse feature maps extracted from encoders should be restored to the original size using a decoder. The encoder–decoder architecture has the disadvantage that the size of the neural network model is increased, because additional parameters are needed to construct the decoder. Therefore, various convolution techniques should be actively applied to maintain the dense estimation performance and reduce the number of parameters required for neural network construction. In addition, the rectified linear unit (ReLU) is mainly used as an activation function when training the CNN. This method is known to be the most general function for solving the vanishing gradient problem in neural networks. However, it is necessary to use another activation function that can solve this problem in neural networks for generating depth maps.

The depth map estimation methods that use a CNN and a single image mainly apply a regression method, because the depth information has a continuous property. However, a depth map prediction by regression can have a large local error, so that it is difficult to estimate the exact depth value. In particular, because the size of the object decreases as its distance increases, the error becomes increasingly large, so that the decreased small objects cannot be identified in the image. Therefore, it is reasonable to estimate the approximate depth value range rather than the exact depth value. In general, the range of depth map datasets is limited, because the depth values are determined by using an infrared projector or a light detection and ranging (LiDAR) system. It is desirable to define a range of depth values as belonging to several classes and perform ordinal regression [14] for each class. In this case, by applying weighting to each distance range and applying a weighting value as the distance increases, it is possible to compensate for errors that increase together with the distance.

Unlike conventional methods, which aim at accurately predicting the 3D depth of a near object and require physical equipment such as an infrared sensor or multi-view camera, the present study proposes the use of an algorithm and an optimized lightweight neural network for quickly generating a depth map using a single image taken in an unstructured indoor or outdoor environment, including under various illumination conditions and camera distances (e.g., a kitchen, living room, classroom, forest, sidewalk, road, tree, or buildings). For estimating a more accurate depth map (1) pixel-based classification is performed to determine the range class to which the approximate depth belongs by using ordinal regression. In addition, (2) various convolution techniques are applied to extract a dense feature map, and (3) the number of parameters is greatly reduced by reducing the number of network layers. To prevent the dying neuron problem of ReLU, which neurons become inactive and only output zero for any input during training, (4) a more flexible model is applied that employs a trainable activation function by modifying ReLU such that it has a nonzero output with a small gradient for negative input. By applying the proposed lightweight neural network algorithm on two datasets, (5) we confirmed that the depth map is quickly generated from a single image and the error based on a comparison with the ground truth is small.

The rest of the paper is organized as follows. In Section 1, we introduce work related to depth information estimation using a single image and a CNN. In Section 2, we describe the main algorithm of the proposed lightweight neural network method. In Section 3, we compare the performance of the proposed method with that of state-of-the-art methods. Finally, in Section 4, our conclusions and future research directions are described.

## 2. Related Work

The conventional depth estimation method requires several multi-view images, and thus depth estimation using a single image, which provides an efficient framework in terms of operations and cost, is a promising alternative. Two methods exist for single image-based depth estimation. The first method is hardware-based and the geometric and physical properties of the camera are used to control the lens focus. Widely used hardware-based methods are defocus-based [15] and multi-focal lens-based depth estimation [16]. Software-based methods estimate depth by applying a learning algorithm, such as machine learning or a deep neural network, to a single image [17]. In our method a depth map is estimated using a single image captured by a general camera, and therefore we explain the related research studies according to the software-based methods. In particular, rather than typical machine learning methods, we explain mainly the recent research trends based on deep learning methods that have been actively studied for depth map estimation using a single image.

In the past few years, deep learning has gained significant attention in diverse fields of study, particularly in computer vision and image processing, and in many applications relevant to such areas. Whereas the depth from a defocus- or multi-focus-based method infers the depth using a single image from a set of multiple image captures, deep learning enables a depth map estimation to be achieved using only a single image. Owing to advances and improvements in a deep neural network, depth estimation using a single 2D image has shown a significant enhancement [11]. Eigen and Fergus [11] constructed two network stacks, one for a coarse network for depth at the global level, and the other for a fine-scale network for depth at the local level; in other words, two deep networks are applied. A joint incorporation of depth-maps from both levels and a depth-map prediction are reasonably achieved.

Tompson et al. [18] proposed hybrid techniques that combine a deep CNN (or ConvNet) and Markov random fields (MRF) and applied algorithms to human body pose estimation and recognition using a single image. Li et al. [19] proposed a method of predicting the depth and surface normal of an image that uses a deep CNN (DCNN), together with post-processing for refining the depth using conditional random fields (CRF). The depth value is refined using CRF at the super pixel level. The authors proposed a mathematical model of the closed form that allows the prediction problem to be solved efficiently. Liu et al. [9] also proposed a depth estimation method based on a CNN and CRF. In [20], it was reported that the depth estimation performance of a CNN is good. In this paper, a matching network using cross entropy was proposed; the trained network contributes to computing the disparities of all the pixels. A depth estimation study conducted without using ground truth data was presented in [21]; however, the approach employed a conventional concept, epipolar geometry constraints, to generate the disparity image by training the network. In the study reported in [13], semi-supervised learning was applied to depth map estimation from a single image. In this method the supervised training used the depth ground truth, as well as unsupervised depth cues. Anirban et al. [22] proposed a neural regression forest (NRF) architecture that combines CNNs with random forests for predicting depth in a continuous domain via regression. The NRF processes a data sample with an ensemble of binary regression trees, and the depth is finally estimated by fusing the individual regression results. The architecture allows all the shallow CNNs to be trained in parallel and efficiently enforces smoothness in the depth estimation results. Laina et al. [23] applied deep residual networks for depth estimation. To improve the output resolution, they presented a new means of efficiently learning feature map upsampling within the network. They also presented a reverse Huber loss that is driven by the value distributions commonly present in-depth maps to achieve the network optimization.

Chakrabarti et al. [24] trained a network to characterize local scene geometry by predicting, at every image location, depth derivatives of different orders, orientations, and scales. The scene depth is then estimated by harmonizing an over-complete set of network predictions, using a globalization procedure. Lee et al. [25] estimated depth maps using a method based on the Fourier frequency domain that utilizes a single image. This method generates multiple depth map candidates by cropping input images using various cropping ratios and a CNN and combines the multiple candidates in the frequency domain to take advantage of their complementary properties.

In recent studies dealing with a depth estimation, Goldman et al. [26] proposed a self-supervised method that uses stereo images for training with a Siamese network, although a single image can also be used for the test process to achieve a monocular depth estimation. The Siamese network architecture consists of two networks, each of which learns to predict disparity maps in a single image. Diaz et al. [27] proposed a simple but effective way to constrain the correlations among categories by seamlessly incorporating metric penalties into the ground-truth labels. This method transforms data labels into a soft probability distribution, pairing well with common category loss functions such as a cross-entropy.

Although numerous studies have been conducted on deep learning to solve the problems involved in computer vision and image processing, few studies on depth estimation using a single image employing the deep learning concept have been reported [28]. According to the studies described above, a single-image-based depth estimation employing the learning technique concept can achieve both efficiency and accuracy in actual practice.

## 3. Design of a Lightweight Neural Network

In this section, we describe the CNN-based neural network and the continuous depth map estimation process that uses the output of the neural network. The proposed neural network consists of two parts. In the first part, a feature map extraction technique is applied to a given image to extract a dense feature map, and then the scale invariance feature is trained using atrous spatial pyramid pooling (ASPP). In the second part, the range of the depth value is estimated by applying ordinal regression [14] to the output of the neural network. Different from [14], the depth value of the actual pixel is assigned to the average of two adjacent labels.

### 3.1. System Architecture

The objective of previously introduced depth estimation neural networks [9,11,13,29,30] was to estimate depth using a DCNN model originally designed for image classification. However, repeated max pooling and convolution constantly reduce the size of the feature map, so that it is difficult to estimate accurately the depth value per pixel. Accordingly, to restore the reduced feature map, it may be possible to apply a multi-scale structure with refinement layers or a multi-layer deconvolution to restore the feature map to its original size. However, these approaches have the disadvantage that they require additional computation and resources because of the increased size of the neural network model.

**Figure 1:**
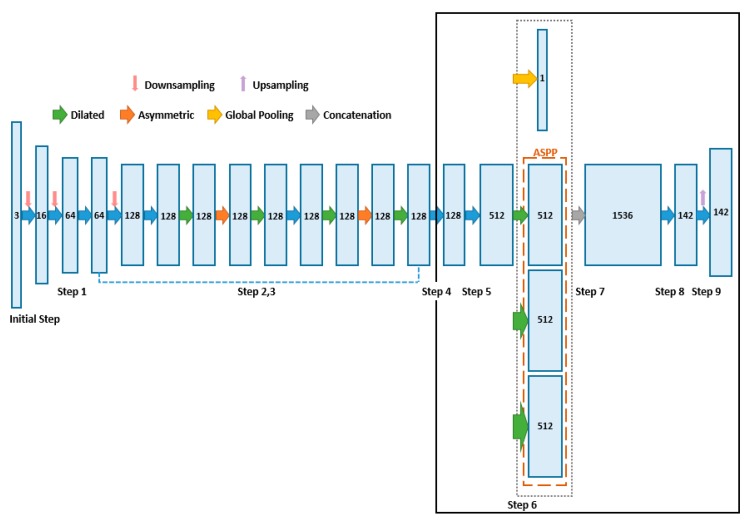
Lightweight efficient neural network architecture. The output size consists of three tuples: number of feature maps, width of a feature map and height of a feature map (142×513×385). The bold black line indicates the newly proposed network structure.

Regarding the compressed structure of a DNN, Wen et al. [31] proposed a structured sparsity learning (SSL) method for normalizing its filters, channels, filter shapes, and layer depths. SSL can be used to learn sparse networks with compressed structures from large-sized DNNs to reduce the operating costs and model size. Huang et al. [32] proposed a simple and effective framework for the learning and pruning of deep models in an end-to-end manner. In this framework, a new type of parameter—namely, a scaling factor—was introduced to scale the output of a particular structure, such as neurons, groups, or residual blocks, by adding a sparsity regularization for these components and using a modified stochastic accelerated proximal gradient (APG). These methods are clearly effective for networks applying general classification, although they are unsuitable for methods that need to estimate a dense depth map. Because the process of predicting a dense depth map is based on the correlation between the features and semantic information, applying this method may degrade the performance. In this study, we designed a lightweight efficient neural network (L-ENet) model, the architecture of which is shown in Figure 1, by modifying the basic ENet [33] model. The original ENet has an encoder–decoder structure designed for semantic image segmentation. It is characterized by a reduction in the number of parameters achieved by using various convolution techniques. To obtain a rich feature from an input image, a convolution is in general performed on a wide receptive field; however, this is inefficient, because the number of parameters required for training increases as the receptive field size increases. ENet uses atrous (dilated) and asymmetric convolution to reduce the overall number of parameters by means of lightened filters, while maintaining a wide receptive field. However, even if the ENet model is lightened, the frequent upsampling process increases the number of parameters of the neural network and requires additional operation time. Therefore, in the proposed L-ENet, the decoder module to restore the feature map size is removed and general convolution operations are replaced with a depth-wise separable convolution. Depth-wise separable convolution separates depth-wise and point-wise convolution to reduce the number of parameters while achieving rich features.

The structure of L-ENet is completed by combining various bottlenecks and consists of 10 steps, as shown in Figure 1. Here the bottleneck module is a combination of several convolution blocks and pooling blocks, as described in detail in Section 3.2. In the Figure 1, the parts in bold line represent the newly proposed L-ENet structure.

**Figure 2:**
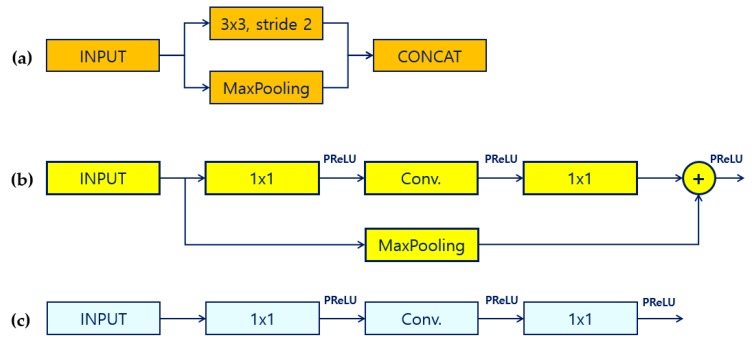
Operation structure of lightweight efficient neural network initial block and bottleneck module. (**a**) Initial module, (**b**) bottleneck module of downsampling operation, and (**c**) bottleneck module of dilated and asymmetric convolution.

- Initial step. Sixteen feature maps of size 256×192 are generated by applying the step shown in Figure 2a to the image input having a size of 512×385.
- Step 1. After downsampling is complete, the bottleneck module shown in Figure 2b is repeated four times to generate 64 feature maps of size 256×96. The bottleneck is explained in detail in Section 3.2.
- Step 2. The bottleneck of the same structure is applied nine (0–8) times. At this time, downsampling, dilated, and asymmetric processes are applied together to generate 128 feature maps of size 64×48.
- Step 3. The bottleneck structures, except bottleneck 2.0, are applied again.
- Steps 4 and 5. The bottleneck is applied to the feature map without upsampling to generate 512 feature maps of size 64×48.
- Steps 6 and 7. Global pooling and ASPP are applied to generate a feature map that is robust against size changes. The operations of Step 6 are processed concurrently in parallel. All the feature map generated using global pooling and ASPP are combined to generate 1537 feature maps of size 64×48.
- Step 8. The bottleneck is applied to the combined feature maps to generate 142 feature maps of size 64×48.
- Step 9. Upsampling is applied to generate 142 feature maps of size 513×385.

In the following section, we explain the structure of the convolution bottleneck and the atrous (dilated), asymmetric convolution method applied in each step. Then the depth-wise separable convolution and regression method used in the proposed L-ENet are described.

### 3.2. Convolution Bottleneck Module

Each bottleneck module consists of several convolution blocks and has pooling blocks inside. The initial module, shown in Figure 2a, constitutes the first part of L-ENet. It performs convolution and pooling on each input and then concatenates each output. The purpose of the initial module is the use of a large stride and max pooling to significantly reduce the input size and apply only a small set of feature maps. Because the visual information is highly spatially redundant, a large input image can be compressed into a more efficient representation through the initial module. Moreover, because network layers of the initial module should not directly contribute to the classification, they act as good feature extractors and only pre-process the input for later portions of a network bottleneck [33].

The L-ENet bottleneck module in Figure 2b,c performs different actions according to the methods listed in the operation of Figure 1. In bottleneck of Figure 2b, the downsampling operation is performed using convolution by adjusting stride. Max pooling is performed in the residual connection to reduce the spatial dimension of the feature map, and then the addition operation is performed. In the dilated and asymmetric operations, bottleneck module of Figure 2c is used to reduce the number of parameters by applying different convolution techniques in the convolution block. The dilated operation means atrous convolution filtering, and the asymmetric operation is a method for applying an asymmetric convolution filter, as shown in Figure 2c. The result of the operation is then output after the addition operation is performed with the result of the residual connection. In the bottleneck module without an operation, the convolution block is designed to perform depth-wise separable convolution. Newly applied depth-wise separable convolution in L-ENet reduces the number of parameters required for convolution and allows faster convolution.

In L-ENet a parametric ReLU (PReLU) [34] is used as an activation function. PReLU is an activation function that modifies ReLU such that it has a nonzero output with a small gradient for negative input. It is defined as

(1) $$f\left(x_{i}\right)=\{\begin{array}{c} x_{i},\;\;x_{i}>0\\ a_{i}x_{i},\;\;x_{i}\leq 0 \end{array},$$

where $x_{i}$ is the input value and $a_{i}$ is the coefficient controlling the gradient of the negative input value. Subscript i of a indicates that the coefficients may vary across the various channels.

PReLU is designed to be controlled with the entire neural network training using a trainable gradient coefficient to solve the problem that the gradient becomes 0 in the existing ReLU, i.e., the dying ReLU problem. Because PReLU is applied on each channel of the neural network, some increases in the parameters’ numbers can occur; however, it is a better option than applying other optimization methods, because it allows more flexible training and overfitting.

The dilated operation in Figure 1 refers to atrous convolution which is a technique that can greatly reduce the number of filter parameters while maintaining the receptive field of the existing convolution. In general convolution, to apply the filter to a larger coverage area, the size of the filter must be increased or the size of the feature map must be reduced by a pooling operation. However, for depth estimation, the reduced feature map must be restored to its original size for dense estimation in pixel units. Therefore, additional computation processes and restoration filters with wide receptive fields are required.

Atrous convolution solves the problem of parameters and the receptive field in different manners. By using the parameters of a general convolution filter as it is and by using one or two holes in the middle, the size of the corresponding receptive field is increased, even if the same filter size is used. Therefore, when atrous convolution is used, the same size filter is maintained, so that the size of the receptive field is enlarged, while the computation amount and the number of parameters are the same. Thus, it becomes possible to handle feature maps of various sizes. The size of the filter depends on the interval (D) at which 0 is inserted in the hole. A general convolution filter is an atrous convolution with D=1. The number after “Dilated” in the operation column in Table 1 indicates the intervals.

**Figure 3:**
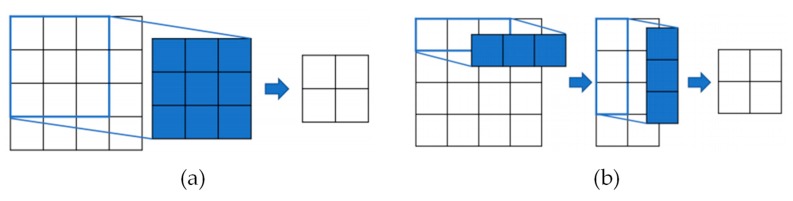
Comparison of a conventional and an asymmetric convolution. (**a**) General conventional convolution operation and (**b**) asymmetric convolution operation.

The asymmetric operation in Figure 1 uses an asymmetric filter as a technique to reduce the number of parameters while maintaining the performance of the existing convolution in the same manner as the atrous convolution. For example, instead of using a 3×3 convolution filter (Figure 3a), convolutions can be performed sequentially using two kernels of 3×1 and 1×3 respectively with the same receptive field, while using considerably fewer parameters (Figure 3b). In this study, a 5×5 asymmetric filter (asymmetric 5) was used.

ASPP in Figure 1 is one of the newly added operations in L-ENet. In ASPP parallel atrous convolutions with three different rates are applied in the input feature map and fused together. As objects of the same class can have different scales in the image, ASPP helps to account for different object scales which can improve accuracy. Unlike existing depth estimation models which use the output of the feature map as it is or attempt to restore the size by using a decoder, L-ENet performs extraction and training of feature maps of various sizes using ASPP. The computation is performed using three filters with intervals of 6, 12, and 18, and cross-channel correlation is trained by convolution.

**Figure 4:**
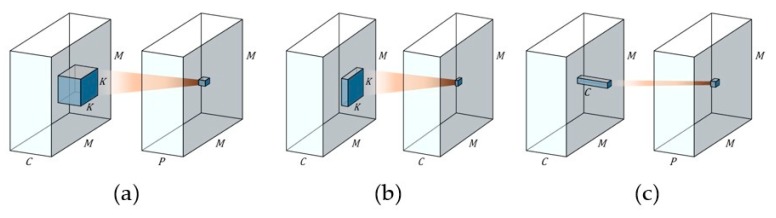
Several convolution methods. (**a**) general convolution, (**b**) depth-wise convolution and (**c**) point-wise convolution.

In the proposed L-ENet, we use the depth-wise separable convolution method [35] to decrease the number of convolution parameters and reduce the correlation between the feature maps generated from each channel. Depth-wise separable convolution is a technique for replacing general convolution (Figure 4a) by combining depth-wise (Figure 4b) and point-wise convolution (Figure 4c). As shown in Figure 4a, if $K^{2}$ filters are applied to M×M sized C channels, one feature map is generated as a result. Therefore, general convolution requires $M^{2}CK^{2}P$ operations to create *P* feature maps. Moreover the $K^{2}CP$ parameters are used for the P channel output. Depth-wise convolution, shown in Figure 4b, performs only spatial convolution without performing channel-wise convolution. Because the filter of $K^{2}$ is performed for each M×M sized channel C, the number of operations becomes $K^{2}CM^{2}$ and the number of parameters becomes $K^{2}C$. Point-wise convolution is a special case where K is 1 in general convolution, because it performs convolution for only a channel using a 1×1 filter, as shown in Figure 4c. Therefore, the number of operations becomes $M^{2}CP$ and the number of parameters required for training is CP. As a result, when general convolution is applied to the same convolution, the number of operations is $M^{2}CK^{2}P$ and the number of parameters is $K^{2}CP$. However, when depth-wise separable convolution is applied, the numbers of operations and parameters are decreased to $K^{2}CM^{2}+M^{2}CP\;\mathrm{and}$ $K^{2}C+CP\;\mathrm{respectively}$. As a result, depth-wise separable convolution separates the depth-wise operation from the point-wise operation which not only drastically reduces the number of parameters, but also weakens the correlation between the spatial-wise and the channel-wise features of the feature map. It is more effective for training contextual features.

### 3.3. Rearrangement of Depth Values

In previous CNN-based depth map estimation studies, depth estimation was treated as a regression problem. The estimation of depth values with continuous properties in a 2D image as a regression leads to the problem that the error between the ground truth and the estimated result becomes larger, because the accuracy of the local depth is decreased. This problem is conspicuously observed in the case of the distant objects rather than the near objects included in an image. This is due to the limitation of using a single 2D image. In this study, we focused on the classification problem by dividing the depth range into several classes, considering the fact that a person looks at an image and predicts the approximate depth range rather than the exact depth value.

**Figure 5:**
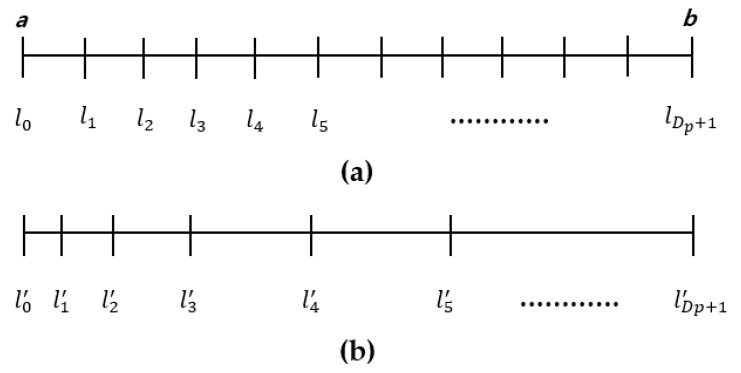
Discretize depth interval [a, b] into Dp+1 subintervals. (**a**) uniformly discretized depth interval and (**b**) discretized depth interval in log space. As the $l_{i}$ label approaches the minimum value *a*, a dense value is reassigned to $l_{i}^{\prime }$. In contrast, as the $l_{i}$ label approaches the maximum value b, a coarse value is reassigned to $l_{i}^{\prime }$.

In L-ENet first the depth value obtained from the LiDAR sensor is divided into Dp+1 depth classes, and when a new test image is input, each pixel of the image is classified into one of Dp+1 depth classes by softmax. After each pixel value is classified into one of the Dp+1 depth classes, the pixel values must be rearranged to have different values for each depth class. This is based on the assumption that a person can make a certain accurate distance prediction for a short distance, but the person’s distance prediction power decreases as the distance increases. In this study, we adopted a spacing-increasing discretization (SID) strategy, as shown in Figure 5, using which the discretization uniformly discretizes a given depth class label in log space. Therefore, the proposed L-ENet is capable of more accurately predicting relatively small and medium depth values and rationally estimating large depth values.

In Equation (2) a and b denote the minimum and maximum depth of ground truth respectively. The depth value of the *a–b* range is divided into Dp+1 intervals according to this equation. If the class label $l_{i}\left(x\right)$ for pixel x is determined as the output value of L-ENet, we recalculate the new depth value $l_{i}^{\prime }\left(x\right)$ for label i and pixel x. Because the size of the object in the image becomes smaller or the distance of the object is infinite as the distance of the object in the image increases, a large error may occur in the inference of the depth. Therefore, Equation (2) allocates a coarse value as the $l_{i}^{\prime }$ label approaches the maximum value b and assigns a dense value as it approaches a. After the class label is recalculated, the depth value of the actual pixel is assigned to the average of two adjacent labels $\frac{l_{i}^{\prime }\left(x\right)\;+l_{i+1}^{\prime }\left(x\right)}{2}$.

(2) $$l_{i}^{\prime }\left(x\right)=e^{\log\left(a\right)+\log\left(\frac{b}{a}\right)\times \frac{l_{i}\left(x\right)}{Dp+1}}.$$

## 4. Experimental Results

To measure the depth estimation performance of the proposed L-ENet, experiments were conducted using the NYU Depth v2 dataset [36], which consists of images captured in indoor environments, and the KITTI dataset [37], which contains images captured in outdoor environments. NYU Depth v2 contains 464 indoor scenes captured by a Microsoft Kinect. The training data consisted of approximately 120 K images evenly selected from 249 scenes and the remaining 694 images were used as test images. As in the study in [14], the input images were resized from 480×640 to 288×384 and then randomly cropped from 288×384 to 257×353 to improve the training speed.

The KITTI dataset contains outdoor data collected from moving vehicles using an RGB camera and an LiDAR sensor. It consists of 61 scenes having a resolution of 375×1241. The training data consisted of approximately 23 K images extracted from 32 scenes and the test data were extracted from 697 test images from 29 scenes. The KITTI dataset consists of various scenes such as ‘city’, ‘residential’, ‘road’, and ‘campus’, allowing these scenes to be distributed evenly across training and test sets. The input image was cropped at a random position before input, where the crop size was 385 × 513, and ground truth was used to perform ordinal regression by setting the maximum value of a class to 80.

In the experiment, we implemented L-ENet using TensorFlow based on Python 3.5. The initial learning rate of the training was 0.0001 and the weight was calculated using the gradient descent method with the momentum set to 0.9. Training was performed with 600 K steps for the KITTI training set and 5 M steps for the NYU Depth v2 training set; the batch size was set to 3. We performed data augmentation using random online transformation, such as scaling, rotation, translation, color conversion, and flipping, as in the study in [11].

For the evaluation of the proposed algorithm, we used the five following commonly used quantitative metrics in depth map estimation:

- root mean squared error (RMSE): $\sqrt{\frac{1}{N}\sum_{i}^{N}{\left({\hat{d}}_{i}-d_{i}\right)}^{2}}$,
- root mean squared log error ($RMSE_{log}$): $\sqrt{\frac{1}{N}\sum_{i}^{N}{\left(\log{\hat{d}}_{i}-\log d_{i}\right)}^{2}}$,
- average relative error (Abs-REL): $\frac{1}{N}\sum_{i}^{N}\frac{\left|{\hat{d}}_{i}-d_{i}\right|}{d_{i}}$,
- accuracy with threshold (δ<t): percentage of $d_{i}$ such that $\max(\frac{{\hat{d}}_{i}}{d_{i}},\frac{d_{i}}{{\hat{d}}_{i}})<t$, where $t=1.25,\;1.25^{2}\;or\;1.25^{3}$.

where the d and $\hat{d}$ are the ground-truth and predicted depth respectively and N is the total number of pixels in the test images.

### 4.1. Performance Evaluation on NYU Depth v2 Dataset

Table 1 shows the performance comparison results of our method for the NYU Depth v2 dataset. To compare the performance, we referred to state-of-the-art methods: Make3D based on Markov random field (MRF) [38], multi-scale convolutional architecture (MSCA) [11], deep convolutional neural fields (DCF) [9], deep neural network embedding focal length (DNNFL) [28], and deep ordinal regression network for monocular depth estimation (DORN) [14].

As shown in Table 1, the proposed method applying NYU Depth v2 is superior to an MRF-based method (Make3D) and deep neural network-based approaches (MSCA [11] and DCF [9]). However, as compared with DORN [14], its performance is 0.01 poorer in terms of δ < 1.25, 0.026 poorer under Abs Rel., and 0.108 poorer in terms of the RMSE. However, although the DORN method requires full-image encoding and an ordinal regression optimizer, it is unsuitable for low-level or depth map measurement systems that require fast computations. In contrast, the proposed method has fewer parameters and operations than DORN, and is thus more suitable for smart phones or embedded systems that require real-time processing because the network size for a depth map estimation is significantly reduced. The numbers of computations and parameters are described in Section 4.3.

### 4.2. Performance Evaluation on KITTI Dataset

For the evaluation on the KITTI dataset; semi-supervised depth map prediction (SSDM) [13]; learn stereo, infer mono (LSIM) [26]; soft ordinal labels (SORD) [27]; and self-supervised relative (SSR) [39] methods were added for the performance comparison. In this experiment, we used ${\mathrm{RMSE}}_{\log}$ instead of RMSE, because the predicted and true values of KITTI images are very large numbers as compared to those of the short distance images of NYU Depth v2. The proposed L-ENet showed a similar or somewhat better performance as compared to those of the other similar algorithms as shown in Table 2. As compared with DORN [14] and SORD [27], the proposed L-ENet showed a performance improvement of approximately 0.033 in terms of δ<1.25 and 0.019-0.027 in terms of ${\mathrm{RMSE}}_{\log}$, but poorer in terms of Abs-REL. DNNFL [28] performed poorly on NYU Depth v2 with near objects, but it showed an improved performance on KITTI data that include remote objects. DNNFL [28] showed a 0.065 improvement over the proposed method in terms of ${\mathrm{RMSE}}_{\log}$. This is because of the errors of the process of performing classification on the approximate depth value rather than the regression of the proposed method. However, the proposed method showed a better performance than DNNFL [28] according to other measurements.

Overall, the methods commonly showed slightly higher errors for the NYU Depth v2 dataset, because the KITTI dataset, which includes images of city roads captured by cameras on moving vehicles, includes not only near objects but also objects at a distance greater than 80 m. For the NYU Depth v2 dataset, the error of the proposed L-ENet is not as large as that of the other deep neural network-based methods, and it can therefore be applied to embedded systems, such as those used in autonomous vehicles that require real-time processing. Thus, the results of the second experiment prove that the proposed L-ENet can be applied to a real-time or embedded system according to the model size and execution time in comparison with other methods, as shown in Table 4.

In addition, to check the robustness of the proposed L-ENets to the noise of the image, we compared its accuracy with threshold with that DORN [14] method using outdoor KITTI dataset by adding 10% Gaussian, Salt & Pepper, and Poisson noises. As shown in Table 3, in both methods, the error increases from the minimum 1% to the maximum 5% for the noise-added images. However, this accuracy is not much lower than other CNN-based methods ([26,27,28]) and can be solved by adding an additional convolution operation to remove noises.

### 4.3. Evaluation of Model Compression

Model size is a very important factor in real-time systems. In this study we compared the model size and the number of parameters of the proposed L-ENet and DORN [14] which showed the best performance among many deep neural network-based methods. Table 4 shows the comparison of the model size for the KITTI dataset containing images in outdoor environments. It can be seen that the proposed method used a significantly decreased model size, that is, by approximately 12.7 times, and the number of parameters was decreased by 12.25 times as compared to those of DORN [14]. In terms of processing time, DORN took 21.6 s for depth estimation based on a CPU, whereas the proposed L-ENet took only 4.3 s in the same system environment. Therefore, if the size of the input image is further reduced and the redundant network is effectively reduced, it is expected that the method can be applied in a smart phone or embedded environment.

### 4.4. Evaluation of Bottleneck Module

Bottleneck modules provide key features for an efficient feature extraction and lightweight DNN structure. A bottleneck module helps diversify the features because it provides several operational paths according to the given operation type. In this study, the existing decoder part is removed based on ENet [33] for an efficient neural network design, and the additional network and bottleneck module are combined, as shown in the bold line of Figure 1. To check the efficiency of the bottleneck module, the bottleneck modules of Steps 4, 5, and 8 were removed from the existing neural network design and replaced with a single convolution layer. We then used the modified neural network design to evaluate the performance under the same conditions as described in Section 4.2. As shown in Table 5, when the bottleneck modules are removed, the performance degrades by approximately 0.035 for δ<1.25 and 0.023 for $RMSE_{log}$ as compared with the existing method. Therefore, the application of the bottleneck module is closely related to the performance of the proposed neural network design.

### 4.5. Ordinal Regression

For the precise estimation of depth values, we applied the ordinal regression method that classifies ordinal depth regions, as does a human being, not exact depth values. To evaluate whether the ordinal regression facilitates an improvement in the performance, the ordinal regression and loss function were replaced with (1) the conventional regression method of [11] and (2) the frequently used mean squared logarithm error (MSLE) method. In addition, we compared the performance of L-ENet without using pooling and ASPP (3) to evaluate the ability of the global pooling and ASPP module. In the experiment, the ordinal regression was applied to the end of the feature extractor instead of the global pooling and ASPP module. Table 6 shows the evaluation results on the KITTI dataset. In the case where the conventional regression method and MSLE were used, the overall performance is poorer than that of other methods relative to the number of training iterations. We can observe that the conventional regression method (1), as well as MSLE (2), make the convergence slow and difficult. In the case where the global pooling and ASPP module were absent (3), the overall performance is poor, because the lost local information is not recovered. However, the proposed ordinal regression (4) facilitates fast convergence and improves the overall performance as compared to other approaches.

**Figure 6:**
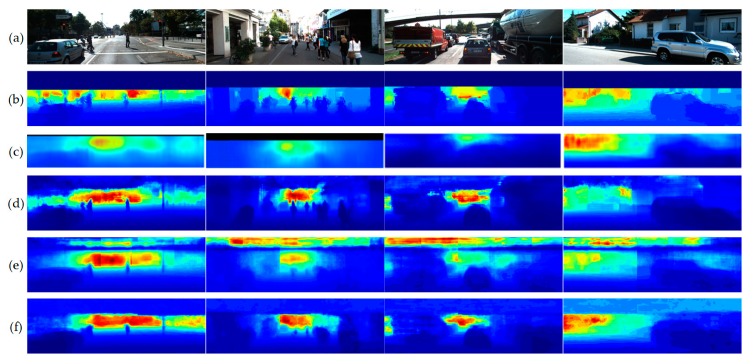
Qualitative comparison of estimated depth-maps on KITTI. (**a**) Input images, (**b**) ground truth, (**c**) multi-scale convolutional architecture [11], (**d**) deep ordinal regression network [14], (**e**) with regression of [11], and (**f**) proposed lightweight efficient neural network.

Figure 6 shows the qualitative depth-map estimation results and a comparison with those of some state-of-the-art methods on KITTI. As shown in Figure 6, the proposed approach estimates the depth map correctly when the background is complex and retains the coarse variation between objects. In addition, common scene layouts and objects—including cars, trees, and pedestrians—are correctly captured from images similar to KITTI ground-truth datasets.

## 5. Conclusions

In this paper, we proposed a new depth estimation method that uses a single image and is based on a lightweight neural network. The method maintains its performance and greatly reduces the number of learning parameters through the application of various convolution techniques. In the conventional methods, because the depth value having a continuous property is solved by a regression problem, the disadvantage exists that the local accuracy is decreased. In addition, when the object in an image becomes small to an undistinguishable level because of the perspective or has an infinite depth value, as in the case of sky, a considerable error is produced. Therefore, the proposed method divides the continuous depth into several labels and classifies each region into classes. At this time, each range is allocated a larger area as the distance becomes greater, thereby compensating for the error that increases with distance.

For real-time depth estimation, this paper introduced depth inference using a lightweight neural network in which various convolution techniques are applied and an approach that is different from existing depth inference methods. Because the existing methods apply many parameters in the deeply constructed neural network model, it was difficult to apply it to a real problem, because a high-performance computing environment was required for practical use. However, if the neural network is designed to be lightweight and reaches a level that can be used in an embedded board, it is meaningful that the high performance of the deep neural network can be demonstrated, regardless of the computing environment.

In future studies, the proposed L-ENet can be extended to capture the fine details of indoor objects and outdoor scenes by including convex, concave, and occlusion edge labels without losing the feedforward network structure. Moreover, we will simplify L-ENet, which can produce depth values closer to those of the ground truth, while reducing the number of layers required for the deep neural network, and test the method in actual practice by applying it to a smartphone or embedded system. Readers can find two application videos from our website, https://cvpr.kmu.ac.kr/ADAS/3D-Depth.html. These videos show that how the proposed method is applied to extracting depth maps of intelligent vehicle and block-based toy 3D modelling applied to smartphones.

## 6. Discussion

The aim of the proposed L-ENet is to accurately predict the 3D depth using a single image taken in an unstructured indoor or outdoor environment instead of physical equipment, such as an infrared sensor or multi-view camera. The main advantage of the proposed method is to reduce the network layers and the number of parameters without losing accuracy. Although the proposed L-ENet algorithm generated an accurate depth map from a single image quickly, it still has a few problems to be solved. Typically, because the upsampling layer consisting of several steps is reduced to one step to improve the processing speed, the prediction error is relatively larger in the near indoor data (e.g., NYU dataset) than outdoor data (e.g., KITTI dataset). Therefore, for dense and accurate depth prediction, it may be necessary to adjust the steps of the upsampling layer according to the application to be used.

## Figures and Tables

**Table 1:** Evaluation result on NYU Depth v2 dataset.

Method	δ<1.25 (Higher Value Is Better)	Abs-REL (Lower Value Is Better)	RMSE (Lower Value Is Better)
Make 3D [38]	0.447	0.349	1.214
MSCA [11]	0.769	0.158	0.641
DCF [9]	0.650	0.213	0.759
DNNFL [28]	0.789	0.151	0.572
DORN [14]	0.828	0.115	0.509
Proposed L-ENet	0.818	0.141	0.617

**Table 2:** Evaluation results on KITTI dataset.

Method	δ<1.25 (Higher Value Is Better)	Abs-REL (Lower Value Is Better)	${\mathrm{RMSE}}_{\log}$ (Lower Value Is Better)
Make 3D [38]	0.601	0.280	0.361
MSCA [11]	0.692	0.190	0.270
DCF [9]	0.647	0.217	0.289
DNNFL [28]	0.893	0.086	0.040
SSDM [13]	0.862	0.113	0.189
SSR [38]	0.840	0.125	0.195
LSIM [26]	0.853	0.113	0.208
SORD [27]	0.931	-	0.124
DORN [14]	0.932	0.072	0.132
Proposed L-ENet	0.965	0.077	0.105

**Table 3:** Accuracy evaluation results on noise-added KITTI dataset.

Method	Gaussian Noise	Salt & Pepper Noise	Poisson Noise
	δ<1.25 (Higher Value Is Better)	δ<1.25 (Higher Value Is Better)	δ<1.25 (Higher Value Is Better)
DORN [14]	0.914	0.891	0.924
Proposed L-ENet	0.933	0.932	0.912

**Table 4:** Comparison of the model size, number of parameters and processing times for the proposed method and a DORN approach using the KITTI dataset.

	DORN [14]	Proposed L-ENet
Model Size	470 MB	37 MB
No. of Params.	117.6 M	9.6 M
Time (s)*	21.6 s	4.3 s

**Table 5:** Evaluation results of bottleneck module using the KITTI dataset.

Variant	δ<1.25 (Higher Is Better)	Abs-REL (Lower Is Better)	${\mathrm{RMSE}}_{\log}$ (Lower Is Better)
Without Bottleneck Module in Step 4-8 of L-ENet	0.930	0.089	0.123
Proposed L-ENet	0.965	0.077	0.105

**Table 6:** Evaluation results with various regression methods on KITTI dataset.

Variant	Iteration	δ<1.25 (Higher Is Better)	Abs-REL (Lower Is Better)	${\mathrm{RMSE}}_{\log}$ (Lower Is Better)
(1) With Regression of [11]	1 M	0.852	0.109	0.171
(2) With MSLE	1 M	0.826	0.158	0.175
(3) Without Pool and ASPP	0.6 M	0.902	0.166	0.119
(4) Proposed L-ENet	0.6 M	0.965	0.077	0.105
